# Real-World Attainment of Guideline-Recommended Lipid Targets in Patients with High and Very High Cardiovascular Risk: A Prospective 12-Month Observational Cohort Study

**DOI:** 10.3390/jcm15145627

**Published:** 2026-07-17

**Authors:** Gabriela Otiman, Ciprian Ilie Rosca, Daniel Dumitru Nisulescu, Abdeldayem Mahmoud, Ana-Maria Pah, Dana Emilia Velimrovici, Cristina Ples, Gheorghe Stoicescu-Hogea

**Affiliations:** 1 Department VI—Cardiology, University Clinic of Internal Medicine and Ambulatory Care, Prevention and Cardiovascular Recovery, “Victor Babeș” University of Medicine and Pharmacy, Eftimie Murgu Square 2, 300041 Timisoara, Romania; otiman.gabriela@umft.ro (G.O.); dana.velimirovici@umft.ro (D.E.V.); gheorghe.stoichescu-hogea@umft.ro (G.S.-H.); 2 Department V—Internal Medicine I, “Victor Babeș” University of Medicine and Pharmacy, Eftimie Murgu Square 2, 300041 Timișoara, Romania; rosca.ciprian@umft.ro; 3 Doctoral School, Department of General Medicine, “Victor Babeș” University of Medicine and Pharmacy, Eftimie Murgu Square 2, 300041 Timisoara, Romania; daniel.nisulescu@umft.ro; 4 Department of Family Medicine, “Victor Babeș” University of Medicine and Pharmacy, Eftimie Murgu Square 2, 300041 Timișoara, Romania; mahmoodemadsalama@gmail.com; 5 Doctoral School, Department of Dental Medicine, “Victor Babeș” University of Medicine and Pharmacy, Eftimie Murgu Square 2, 300041 Timisoara, Romania; cristina.stoichescu-hogea@umft.ro

**Keywords:** LDL-cholesterol, non-HDL-cholesterol, cardiovascular risk, lipid-lowering therapy, target attainment, therapeutic inertia, statin, ezetimibe, PCSK9 inhibitor, SCORE2, real-world evidence

## Abstract

**Background/Objectives:** European guidelines recommend increasingly stringent, risk-stratified low-density lipoprotein cholesterol (LDL-C) targets, but real-world attainment remains suboptimal. We evaluated 12-month lipid-target attainment and changes in model-derived cardiovascular risk estimates in an ambulatory dyslipidaemia cohort. **Methods:** In this prospective, single-centre observational study, 101 consecutive adults were assessed at baseline and after at least 12 months of routine-care lipid-lowering therapy. Patients were classified as moderate/low, high, or very high cardiovascular risk. The primary endpoint was attainment of the risk-specific LDL-C target at 12 months versus baseline. Paired changes were tested using the McNemar exact and Wilcoxon signed-rank tests. Multivariable logistic regression assessed correlates of target attainment, with Firth penalisation used because of sparse outcomes in the higher risk strata. **Results:** LDL-C target attainment increased from 6.0% to 43.0%, and non-HDL-C attainment from 7.0% to 51.5% (both *p* < 0.001). At 12 months, 89% of moderate/low-risk patients reached the LDL-C target, compared with 9% of high-risk and 23% of very-high-risk patients (*p* < 0.001). Higher risk category was strongly associated with non-attainment, although the odds ratios were imprecise because of sparse events. Only one patient received a high-intensity statin and three received a PCSK9 inhibitor, despite 63% of the cohort being at high or very high risk. Model-derived risk scores decreased, partly as a mathematical consequence of updated lipid inputs. Therapy was well tolerated. **Conclusions:** Lipid control improved substantially, but target attainment remained poorest in patients at greatest cardiovascular risk. Earlier treatment intensification, structured adherence assessment, and more consistent use of combination therapy may reduce this implementation gap.

## 1. Introduction

Atherosclerotic cardiovascular disease (ASCVD) remains the leading cause of mortality worldwide, and a large body of mechanistic, genetic and interventional evidence has established low-density lipoprotein cholesterol (LDL-C) as a causal driver of atherogenesis. The relationship between absolute LDL-C reduction and the relative reduction in major adverse cardiovascular events is continuous, log-linear and apparently independent of the mechanism by which LDL-C is lowered, which provides the rationale for the principle that, for LDL-C, “lower is better” and “earlier is better” [1,2].

On this basis, the 2019 European Society of Cardiology (ESC) and European Atherosclerosis Society (EAS) guidelines for the management of dyslipidaemias, reaffirmed in the 2021 ESC prevention guidelines, introduced progressively stricter, risk-stratified LDL-C goals: below 1.4 mmol/L (55 mg/dL) for very-high-risk individuals, below 1.8 mmol/L (70 mg/dL) for high-risk individuals, and below 3.0 mmol/L (116 mg/dL) for those at moderate or low risk. Each goal is accompanied by a requirement for at least a 50% reduction from baseline in the two highest risk strata. Non-HDL-C, which captures the cholesterol carried by all apolipoprotein-B-containing lipoproteins, was endorsed as a co-primary treatment target, with thresholds of 2.2 mmol/L (85 mg/dL) and 2.6 mmol/L (100 mg/dL) for very-high- and high-risk patients, respectively [3,4].

Despite this clear and increasingly ambitious guidance, a persistent and well-documented gap separates guideline recommendations from everyday clinical practice. Large multinational registries, including DA VINCI, EUROASPIRE and SANTORINI, have repeatedly demonstrated that only a minority of high- and very-high-risk patients achieve their recommended LDL-C goal, and that the shortfall is greatest precisely in those at highest absolute risk—the population in which the expected absolute benefit of treatment is largest. The drivers of this gap are multifactorial and include therapeutic inertia, under-prescription of high-intensity statins and combination therapy, suboptimal adherence, and incomplete implementation of newer agents such as ezetimibe and proprotein-convertase-subtilisin-kexin-type-9 (PCSK9) inhibitors [5,6,7,8,9,10]. Recent European evidence reinforces these implementation barriers: risk-category assignment has been associated with failure to achieve therapeutic targets in patients with type 2 diabetes, while the Italian ITACARE-P network documented persistent therapeutic inertia in secondary cardiovascular prevention despite off-target LDL-C values [11,12].

Most of the available real-world evidence derives from cross-sectional registries or from settings within Western and Northern Europe. Prospective, longitudinal data describing the trajectory of lipid control and cardiovascular risk under structured, guideline-directed therapy in Central and Eastern European ambulatory populations remain comparatively scarce. Furthermore, few studies have combined contemporary risk-stratification instruments—SCORE2, the lifetime LIFE-CVD and DIAL models, the SMART and SMART-REACH residual-risk scores, and the recently introduced PREVENT equations—with serial lipid measurements to characterise how both lipid targets and quantitative risk estimates evolve over a full year of treatment [13,14,15,16,17,18].

Accordingly, this prospective study evaluated 12-month attainment of risk-stratified LDL-C and non-HDL-C targets, changes in validated cardiovascular risk estimates, and clinical determinants of LDL-C target non-attainment in a Central and Eastern European ambulatory cohort.

## 2. Materials and Methods

### 2.1. Study Design and Population

We conducted a prospective, single-centre, observational cohort study at a university-affiliated cardiovascular-prevention outpatient clinic in Timișoara, Romania, following a protocol that was finalised and ethically approved before the enrolment of the first participant. Consecutive adults (≥18 years) referred for the management of dyslipidaemia were enrolled prospectively between 1 January 2024 and 31 December 2024, and 101 patients with a complete baseline lipid profile and at least 12 months of subsequent follow-up were included. Each participant was followed prospectively and assessed at three pre-specified time points defined a priori in the protocol: baseline, immediately before the initiation or intensification of lipid-lowering therapy (T0); a short-term reassessment 6–12 weeks after each therapeutic change (T1); and a long-term reassessment after a minimum of one year of treatment (T2). Demographic, clinical, biochemical and therapeutic data were collected prospectively at each scheduled visit on standardised case-report forms. The study was conducted in accordance with the Declaration of Helsinki and the principles of Good Clinical Practice; the study protocol was reviewed and approved by the institutional ethics committee prior to the enrolment of the first participant, and every participant provided written informed consent before any study-related procedure was undertaken. The database was fully de-identified prior to analysis.

### 2.2. Clinical Assessment and Cardiovascular Risk Stratification

At each visit, demographic data, anthropometry (body-mass index, waist circumference), lifestyle factors (smoking status, alcohol intake, sedentary behaviour, self-reported stress), comorbidities and a complete biochemical lipid panel were recorded. Comorbidities captured included diabetes mellitus and its duration and target-organ damage, established ASCVD (coronary, cerebrovascular, peripheral arterial or polyvascular disease), arterial hypertension, chronic kidney disease staged by the CKD-EPI estimated glomerular filtration rate (eGFR) and urinary albumin-to-creatinine ratio, left-ventricular hypertrophy, heart failure, atrial fibrillation, and a range of risk-modifying conditions (chronic immune-mediated inflammatory disease, obstructive sleep apnoea/chronic obstructive pulmonary disease, hepatic steatosis, and others).

Patients were stratified into three cardiovascular risk categories in accordance with the 2021 ESC prevention framework. The very-high-risk category comprised patients with documented ASCVD or with diabetes mellitus and target-organ damage; the high-risk category comprised patients with diabetes mellitus, markedly elevated single risk factors, moderate-to-severe chronic kidney disease, or a SCORE2 estimate within the high-risk band for their age stratum; and the remaining patients were classified as moderate/low risk. Quantitative risk was further characterised using the SCORE2/SCORE2-Diabetes algorithms, the lifetime LIFE-CVD and DIAL models, the SMART and SMART-REACH residual-risk scores for patients with established disease, and the PREVENT equations [13,14,15,16,17,18].

### 2.3. Lipid Targets and Endpoints

At every visit, a complete fasting serum lipid panel (total cholesterol, LDL-C, HDL-C, non-HDL-C, remnant cholesterol and triglycerides) was measured. For the primary analysis these values were classified against pre-specified, guideline-anchored category boundaries defined a priori in the study codebook so as to preserve the clinically decisive treatment thresholds of the 2019 ESC/EAS guidelines and to provide a threshold-anchored endpoint robust to assay-level measurement variability. Target attainment was operationalised against the absolute LDL-C threshold, and the additional guideline co-criterion of a ≥50% reduction from baseline was not formally evaluated in the primary endpoint. The primary endpoint was the proportion of patients achieving the risk-stratified LDL-C target at T2 compared with T0, defined as <55 mg/dL for very-high-risk, <70 mg/dL for high-risk, and <116 mg/dL for moderate/low-risk patients. Secondary endpoints comprised non-HDL-C target attainment (<85 mg/dL for very-high-risk and <100 mg/dL for high-risk patients), the ordinal shift across lipid strata, changes in validated cardiovascular risk scores between T0 and T2, and the safety profile of therapy assessed by serial transaminases and creatine kinase.

### 2.4. Lipid-Lowering and Adjunctive Therapy

Management followed a structured, guideline-directed framework comprising lifestyle counselling and pharmacological therapy individualized to risk category and baseline LDL-C, applied within routine ambulatory practice. Consistent with the real-world, observational design, all individual prescribing decisions—including statin intensity and the initiation of combination or non-statin therapy—remained at the discretion of the treating physician rather than being mandated by protocol. The resulting difference between the guideline-directed treatment intensity that the framework intended and the intensity actually delivered was, therefore, not a protocol deviation but a pre-specified object of study, analysed explicitly as a determinant of target attainment (Section 3.5). Statin type and intensity, ezetimibe, fibrate, PCSK9 inhibitor and omega-3 fatty-acid use were recorded prospectively at each visit, together with optimal antihypertensive therapy, antiplatelet agents, and glucagon-like-peptide-1 receptor agonists or sodium-glucose co-transporter-2 inhibitors where indicated.

### 2.5. Statistical Analysis

Continuous variables are summarised as mean ± standard deviation or median (interquartile range), according to distribution assessed using the Shapiro–Wilk test; categorical variables are presented as counts and percentages. Paired changes in dichotomous target-attainment endpoints between T0 and T2 were evaluated using the McNemar exact test, and ordinal shifts in lipid and risk-score categories using the Wilcoxon signed-rank test. Between-group comparisons used the Mann–Whitney U test or the chi-square/Fisher exact test, as appropriate. Independent correlates of LDL-C target attainment at T2 were examined using multivariable logistic regression. The model included age (per year), sex, assigned cardiovascular risk category (moderate/low risk as the reference), and statin–ezetimibe combination therapy. Multicollinearity was assessed using variance inflation factors; all values were <2.0, indicating no problematic collinearity. Diabetes mellitus was not entered simultaneously with risk category because diabetes is an input criterion for the higher risk strata and would therefore introduce structural collinearity; its association with attainment was examined separately in univariable analysis. Because sparse target-attainment events in the high- and very-high-risk strata produced quasi-complete separation in the conventional maximum-likelihood model, the analysis was repeated using Firth penalized-likelihood logistic regression to obtain more stable estimates. Results are expressed as odds ratios (OR) with 95% confidence intervals (CI). All tests were two-sided, and *p* < 0.05 was considered statistically significant. Analyses were performed in Python 3.12.13 (Python Software Foundation; https://www.python.org/, accessed on 1 July 2026), with pandas version 3.0.4 (https://pandas.pydata.org/, accessed on 1 July 2026), SciPy version 1.18.0 (https://scipy.org/, accessed on 1 July 2026), and statsmodels version 0.14.6 (https://www.statsmodels.org/, accessed on 1 July 2026). Firth penalized-likelihood logistic regression was performed using R version 4.6.1 (R Foundation for Statistical Computing, Vienna, Austria; https://www.r-project.org/, accessed on 1 July 2026) and the logistf package version 1.26.1 (https://CRAN.R-project.org/package=logistf, accessed on 1 July 2026).

## 3. Results

### 3.1. Baseline Characteristics

The cohort comprised 101 patients with a mean age of 58.2 ± 7.1 years, of whom 60 (59.4%) were women and 64 (63%) lived in an urban environment. Diabetes mellitus was present in 39 patients (39%) and established ASCVD in 23 (23%); almost the entire cohort had treated or untreated arterial hypertension at baseline (99/101). A family history of premature cardiovascular disease was reported by 44 patients (44%), 37 (37%) were active smokers, and 66 (65%) were sedentary. Only 10 patients (10%) were statin-naïve at enrolment. Stratification yielded 32 very-high-risk, 32 high-risk and 37 moderate/low-risk patients. Baseline characteristics are summarised in Table 1.

### 3.2. Primary Endpoint: LDL-C and Non-HDL-C Target Attainment

**Figure 1:**
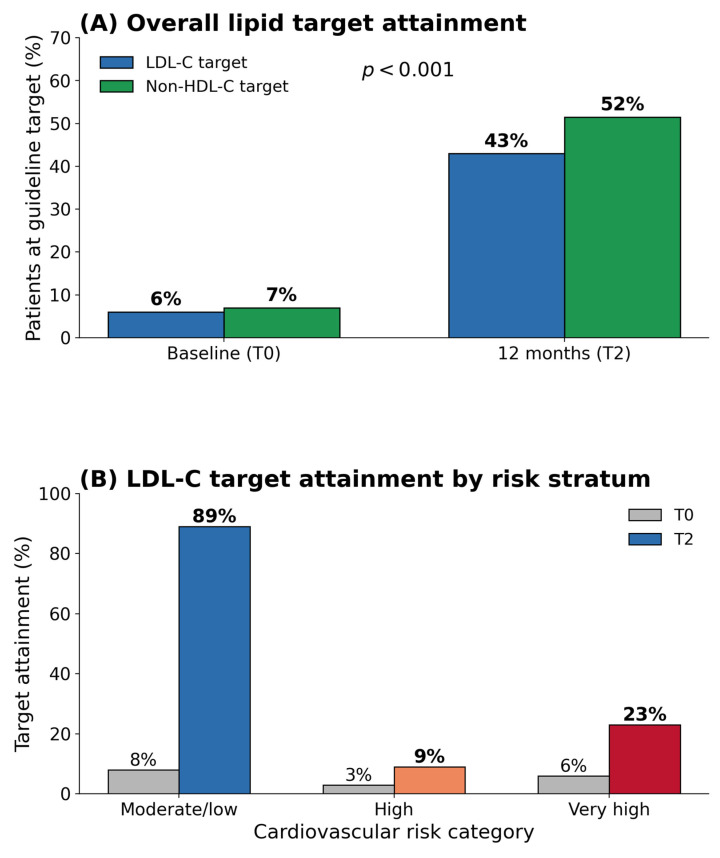
Lipid target attainment. (**A**) Overall proportion of patients achieving the risk-stratified LDL-C and non-HDL-C targets at baseline (T0) versus 12 months (T2); both increased significantly (*p* < 0.001, McNemar exact test). (**B**) LDL-C target attainment at 12 months stratified by cardiovascular risk category, demonstrating the inverse relationship between target strictness and attainment, which was not strictly monotonic across the high- and very-high-risk strata. T0, baseline; T2, ≥1-year follow-up.

Guideline-directed therapy produced a marked overall improvement in lipid control. The proportion of patients meeting their risk-stratified LDL-C target rose more than six-fold, from 6.0% at baseline to 43.0% at 12 months (38 patients reached the target, 1 missed it; McNemar exact *p* < 0.001). Non-HDL-C target attainment increased in parallel, from 7.0% to 51.5% (45 reached, 1 missed; *p* < 0.001). These primary findings are illustrated in Figure 1A.

**Figure 2:**
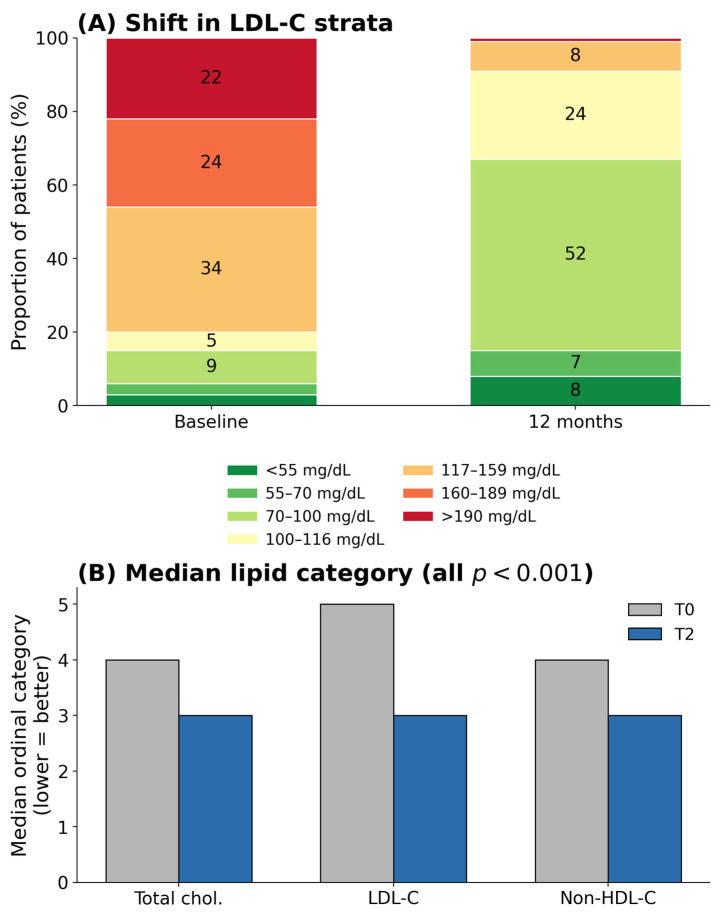
Shift in lipid strata under therapy. (**A**) Stacked distribution of LDL-C categories at baseline and 12 months, showing a pronounced redistribution toward lower (greener) strata. (**B**) Median ordinal lipid category for total cholesterol, LDL-C and non-HDL-C at T0 versus T2; all comparisons *p* < 0.001 (Wilcoxon signed-rank test). Lower categories denote lower, more favourable concentrations.

The ordinal distribution of LDL-C categories shifted substantially toward lower values, with the median LDL-C stratum improving from category 5 (117–159 mg/dL) to category 3 (70–100 mg/dL) (Wilcoxon *p* < 0.001). Concordant, statistically significant downward shifts were observed for total cholesterol and non-HDL-C (both *p* < 0.001) and, more modestly, for remnant cholesterol (*p* = 0.002). These distributional changes are shown in Figure 2.

### 3.3. The Inverse Risk Gradient in Target Attainment

The most clinically salient finding emerged on stratification by risk category. Despite a strict target requirement, moderate/low-risk patients reached their LDL-C goal in 89% of cases at T2, whereas only 23% of very-high-risk and a mere 9% of high-risk patients did so (*p* < 0.001; Figure 1B). A broadly similar pattern was observed for non-HDL-C (89% moderate/low, 38% high, 22% very high). Target attainment was thus inversely related to the strictness of the risk-based target, such that the patients with the greatest potential absolute benefit from intensive lipid lowering were among the least likely to reach their goal. The gradient was not strictly monotonic, however: very-high-risk patients attained their LDL-C target somewhat more often than high-risk patients (23% vs. 9%) despite facing the stricter threshold—a pattern considered further in the Discussion. Stratified attainment data are summarised in Table 2.

### 3.4. Predictors of LDL-C Target Attainment

Assigned cardiovascular risk category was the principal independent correlate of LDL-C target attainment. The conventional maximum-likelihood model showed quasi-complete separation and generated unstable estimates for the higher risk strata; therefore, the Firth penalized-likelihood model was used for primary interpretation. Relative to the moderate/low-risk group, high-risk status was associated with lower odds of target attainment (OR 0.012, 95% CI 0.002–0.06; *p* < 0.001), as was very-high-risk status (OR 0.034, 95% CI 0.009–0.12; *p* < 0.001). These odds ratios should be interpreted primarily as indicators of the direction and strength of association rather than as precise estimates of effect size. Although Firth penalisation reduces small-sample bias caused by separation, the confidence intervals remain wide relative to the point estimates, and the results are sensitive to the small number of attainment events in the higher risk strata. Age, sex, and statin–ezetimibe combination therapy were not independently associated with attainment (all *p* > 0.05). In univariable analysis, patients with diabetes were less likely to reach the target than those without diabetes (18% vs. 58%; *p* < 0.001); however, diabetes overlapped substantially with assignment to the higher risk strata and was, therefore, not included simultaneously with risk category. The multivariable findings are summarised in Table 3.

### 3.5. Therapeutic Patterns and Inertia

**Figure 3:**
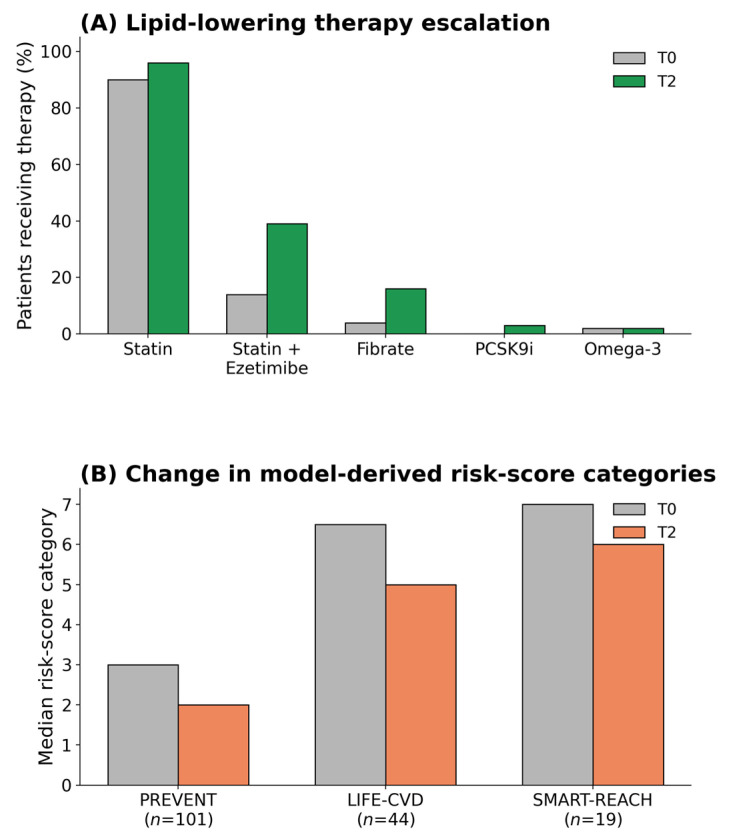
Therapy escalation and change in model-derived risk scores. (**A**) Proportion of patients receiving each lipid-lowering therapy class at baseline versus 12 months. (**B**) Median category of the PREVENT, LIFE-CVD and SMART-REACH risk scores at T0 versus T2. These are recalculated model outputs and should not be interpreted as observed reductions in cardiovascular events. PCSK9i, proprotein-convertase-subtilisin-kexin-type-9 inhibitor.

By 12 months, 97 patients (96%) were receiving a statin and statin–ezetimibe combination therapy had been adopted in 39 (39%), up from 14 at baseline. A high-intensity regimen was defined a priori, in accordance with the 2019 ESC/EAS guidelines, as atorvastatin ≥ 40 mg or rosuvastatin ≥ 20 mg daily—that is, a regimen with an expected LDL-C reduction of at least 50%. By this definition, treatment intensity remained strikingly conservative relative to the risk profile of the cohort: only a single patient was prescribed a high-intensity statin regimen, and a PCSK9 inhibitor was used in only three patients, despite 64 of 101 patients (63%) being at high or very high risk. The substantial category-level improvement in LDL-C control was, therefore, achieved predominantly through the initiation and up-titration of low- and moderate-intensity statins—almost the entire cohort was statin-treated by T2—and through ezetimibe add-on in 39% of patients, rather than through uptake of high-intensity statin therapy. This pattern is itself a direct expression of the therapeutic inertia the study set out to quantify: the moderate gains achievable with low- and moderate-intensity regimens were sufficient to move many moderate/low-risk patients across their lenient threshold, but left the high- and very-high-risk patients—who require the larger reductions delivered by high-intensity or combination therapy—short of their stricter goals. Fibrates were used in 16 patients and omega-3 fatty acids in 2. Among very-high-risk patients who failed to reach the LDL-C target, 7 of 25 (28%) also exhibited persistently elevated triglycerides at T2, pointing to a residual, partly atherogenic-dyslipidaemia-driven risk. The escalation of lipid-lowering therapy is depicted in Figure 3A.

### 3.6. Cardiovascular Risk-Score Trajectories

Improved lipid values were accompanied by lower recalculated cardiovascular risk-score estimates. The PREVENT risk category fell significantly across the whole cohort (median category 3 to 2; *p* < 0.001), as did the lifetime LIFE-CVD estimate (*n* = 44; median 6.5 to 5.0; *p* < 0.001), and among patients with established disease, the SMART and SMART-REACH residual-risk scores also fell (*p* = 0.034 and *p* = 0.014, respectively). The DIAL lifetime estimate in patients with diabetes showed a non-significant trend toward improvement (*n* = 13; *p* = 0.38), likely reflecting the limited statistical power in this subgroup. Because total cholesterol and LDL-C/non-HDL-C are direct inputs to several of these algorithms, part of the observed score change is mathematically expected when updated lipid values are entered. These findings therefore demonstrate changes in modelled risk estimates and responsiveness of the calculators to modified inputs; they do not independently demonstrate treatment benefit, causal risk reduction, or fewer cardiovascular events. These trajectories are summarised in Figure 3B.

### 3.7. Safety

Therapy was well tolerated. Transaminase elevations were recorded in seven patients (alanine aminotransferase) and six (aspartate aminotransferase) at T2, all of modest magnitude, and a single patient showed a creatine-kinase elevation at the short-term reassessment. No patient discontinued therapy for a safety reason during follow-up, and high-sensitivity C-reactive protein did not change significantly (*p* = 0.10).

## 4. Discussion

In this prospective 12-month observational cohort, lipid control improved substantially: overall LDL-C target attainment increased from 6.0% to 43.0%, and non-HDL-C attainment from 7.0% to 51.5%, with a reassuring safety profile. The principal finding was the persistent risk-stratified attainment gap. At 12 months, 89% of moderate/low-risk patients reached the LDL-C goal, compared with 9% of high-risk and 23% of very-high-risk patients. Thus, the patients with the greatest expected absolute benefit remained the least likely to achieve their recommended target. These findings are consistent with the 2019 ESC/EAS and 2021 ESC prevention framework [3,4] and extend prior cross-sectional registry evidence by providing a longitudinal, within-patient assessment of lipid targets and contemporary cardiovascular risk estimates in a Central and Eastern European ambulatory cohort.

### 4.1. Comparison with European Real-World Registries

Our overall attainment figures and, in particular, the risk-stratified shortfall align closely with major contemporary European registries while extending them prospectively. In the pan-European DA VINCI study of 5888 patients, only about one-third (33%) of very-high-risk patients reached the 2019 LDL-C goal, and attainment with moderate-intensity statin monotherapy—the dominant regimen—was substantially lower [6]. The geographically proximate DA VINCI Central and Eastern European sub-analysis, which included Romanian sites, reported even wider gaps and heavier reliance on statin monotherapy than the Western European average [7]. Our very-high-risk attainment of 23% is consistent with the lower end of this regional range. Likewise, a contemporary European cohort of patients with type 2 diabetes showed that achievement of therapeutic targets was strongly influenced by assigned cardiovascular risk category, supporting the diabetes-related shortfall observed in our cohort [11].

The multinational SANTORINI study of more than 9000 high- and very-high-risk patients reached the same central conclusion from a different angle: median LDL-C levels in both the high- and very-high-risk strata (93 and 78 mg/dL, respectively) remained well above goal, only around one-fifth of patients achieved the 2019 LDL-C target, and combination therapy was used in a minority—mirroring our finding that the statin–ezetimibe combination reached only 39% and PCSK9 inhibition only 3% of patients, despite 63% of the cohort being at high or very high risk [8]. The EUROASPIRE V surveys, spanning both secondary- and primary-prevention populations across 27 and 16 European countries respectively, similarly documented that roughly half of very-high-risk individuals failed to reach recommended cholesterol goals and that risk-factor control had plateaued—an observation our prospective data reinforce at the level of the individual patient trajectory [9,10].

A simulation study applying the 2019 thresholds to a nationwide post-myocardial-infarction cohort reached a parallel conclusion at the mechanistic level: for the majority of very-high-risk patients, the goal of <55 mg/dL is mathematically unachievable with statin monotherapy alone, and combination therapy is required a priori [19]. Our data are the practical corollary of that simulation—when escalation does not occur, the predicted shortfall materialises, and it does so most severely in the highest risk group.

### 4.2. Mechanisms of the Inverse Attainment Gradient

Two complementary mechanisms account for this gap. The first is arithmetic. Very-high- and high-risk patients face far stricter absolute thresholds (<55 and <70 mg/dL) than moderate/low-risk patients (<116 mg/dL); because the proportional LDL-C reduction achievable with a given regimen is broadly similar across patients, an identical relative reduction leaves high-risk patients above goal far more often. The Cholesterol Treatment Trialists’ meta-analyses establish that each 1 mmol/L reduction in LDL-C lowers major vascular events by approximately one-fifth, irrespective of baseline risk, so the absolute benefit forgone when the highest risk patients miss target is correspondingly the largest [5,20,21]. This arithmetic also explains why the gradient need not be perfectly monotonic: that very-high-risk patients reached their stricter target more often than high-risk patients (23% vs 9%) most plausibly reflects more intensive treatment and closer follow-up in patients with established atherosclerotic disease, whose secondary-prevention status prompts earlier statin use and more frequent review, partially offsetting their stricter threshold. The modest number of patients within each stratum also limits the precision of these stratum-specific estimates and counsels caution in over-interpreting the exact ordering.

The second, and more remediable, mechanism is therapeutic inertia. This phenomenon has recently been characterised in detail by the Italian ITACARE-P network, which documented that a substantial proportion of very-high-risk patients in secondary prevention remained on unchanged, insufficiently intensive lipid-lowering therapy despite persistently off-target LDL-C—closely mirroring the conservative prescribing pattern we observed [12]. The guideline-endorsed escalation pathway—high-intensity statin, then ezetimibe, then a PCSK9 inhibitor—was implemented only partially in our cohort: high-intensity statin therapy was prescribed to a single patient, ezetimibe combination to 39%, and a PCSK9 inhibitor to only three, despite robust outcome evidence for each escalation step. The IMPROVE-IT trial demonstrated that adding ezetimibe to statin therapy after acute coronary syndrome reduced LDL-C to a median of 54 mg/dL and significantly lowered cardiovascular events [22]; the FOURIER and ODYSSEY OUTCOMES trials showed that PCSK9 inhibition on a statin background drives LDL-C to roughly 30–50 mg/dL with further event reduction [23,24]; and CLEAR Outcomes and the ORION inclisiran programme have broadened the non-statin armamentarium, including for statin-intolerant patients—a phenotype with a meta-analytic prevalence of only around 9%, far below the rate often invoked to justify under-treatment [25,26,27]. The secondary-prevention meta-analysis by Koskinas et al. confirms that the benefit of non-statin LDL-lowering tracks the magnitude of LDL-C reduction, reinforcing that the escalation steps omitted in our cohort are precisely those with proven incremental value [28].

In addition to lowering LDL-C, statins exert several biologically plausible pleiotropic effects that may contribute to cardiovascular protection, including attenuation of vascular inflammation, improvement of endothelial function and nitric-oxide bioavailability, stabilization of atherosclerotic plaques, and modulation of platelet activation and coagulation pathways [29]. These mechanisms provide additional biological rationale for timely initiation and appropriate intensification of statin therapy. Nevertheless, the present study did not measure inflammatory, endothelial, plaque, or haemostatic endpoints, and it cannot quantify the clinical contribution of these non-lipid effects; they should therefore be viewed as complementary mechanisms rather than substitutes for achieving guideline-recommended LDL-C targets.

The Firth penalized multivariable analysis was directionally consistent with this interpretation: high-risk (OR 0.012, 95% CI 0.002–0.06) and very-high-risk status (OR 0.034, 95% CI 0.009–0.12) were associated with lower odds of target attainment relative to moderate/low risk, whereas age, sex, and combination therapy were not independently associated. However, the magnitude of these odds ratios must be interpreted cautiously. They are primarily markers of association in a sparse dataset, not precise estimates of effect size, and they should not be interpreted causally. Firth penalisation addresses separation and reduces finite-sample bias but does not eliminate the substantial uncertainty created by the small number of attainment events. The univariable association between diabetes and non-attainment (18% vs. 58%) was largely explained by its overlap with the higher risk strata, suggesting that the stricter target combined with insufficient treatment intensity, rather than diabetes itself, may have been the operative barrier [30].

### 4.3. Residual Risk Beyond LDL-C

The persistence of elevated triglycerides in a subset of very-high-risk patients who failed to reach the LDL-C target (7 of 25) points to a residual, partly atherogenic-dyslipidaemia-driven component of risk that statin monotherapy addresses incompletely. A growing body of evidence positions apolipoprotein B and remnant cholesterol as treatment-refractory residual-risk markers: apolipoprotein B captures the total atherogenic particle number that LDL-C alone may underestimate [31,32], remnant cholesterol predicts ASCVD independently of LDL-C and apolipoprotein B [33], and triglyceride-rich lipoproteins are increasingly recognised as causal contributors to atherogenesis [34]. That our cohort showed only a modest reduction in the remnant-cholesterol category (*p* = 0.002) relative to the marked LDL-C shift highlights that comprehensive risk reduction in the highest risk patients may require attention to non-LDL atherogenic lipoproteins, in addition to intensified LDL-C lowering.

### 4.4. Risk-Score Trajectories and Clinical Implications

The statistically significant decreases in PREVENT, LIFE-CVD, SMART and SMART-REACH should be interpreted as changes in recalculated model outputs rather than as independent evidence of cardiovascular risk reduction. LDL-C, total cholesterol and other modifiable variables are explicit inputs to several of these equations; consequently, part of the observed improvement is mathematically expected by construction when more favourable follow-up values are entered. The consistency across differently constructed instruments indicates that the updated clinical profile generated lower modelled estimates, but it does not demonstrate a causal treatment effect or a reduction in observed cardiovascular events. These tools may nevertheless be useful for communicating expected benefit and supporting shared decision-making and adherence, because the presentation of cardiovascular risk can materially influence patient perceptions and treatment preferences [18,35].

From an implementation standpoint, our findings support earlier and more decisive up-titration and combination therapy in high- and very-high-risk patients—potentially including an upfront fixed-dose statin–ezetimibe strategy rather than prolonged sequential monotherapy—and wider, guideline-concordant use of PCSK9-directed agents and other non-statin therapies when targets remain unmet, in line with international expert recommendations for the post-acute-coronary-syndrome setting and beyond [36]. Structured follow-up, explicit assessment of adherence, and decision-support prompts for escalation may be particularly useful in the highest risk strata.

Any strategy of treatment intensification should also remain clinically monitored and individualized. The major outcomes of intensive non-statin LDL-C lowering trials cited above did not identify a broad safety signal that outweighed cardiovascular benefit [23,24], but the clinical implications of very low LDL-C concentrations remain an evolving area of investigation. In an observational cohort of anticoagulated patients with venous thromboembolism, LDL-C below 70 mg/dL was associated with a higher risk of bleeding, particularly non-major bleeding and haematomas [37]. This finding is hypothesis-generating and cannot establish that lipid lowering caused bleeding; the study population, concomitant anticoagulation, and clinical context differ substantially from those of our ambulatory cohort. It should therefore not be generalized as a reason to withhold guideline-directed lipid-lowering therapy, but it supports individualized bleeding-risk assessment and closer surveillance in patients receiving concomitant anticoagulant or antithrombotic treatment.

### 4.5. Strengths and Limitations

Beyond confirming the target-attainment shortfall documented in cross-sectional registries, the principal incremental contribution of this study is its prospective, individual-level characterisation of how risk-stratified lipid targets and five contemporary risk scores evolve over a full year of structured treatment in a Central/Eastern European ambulatory setting, where such longitudinal data remain scarce. Its strengths include the prospective three-time-point design, granular phenotyping, simultaneous use of five contemporary risk instruments, and complete one-year follow-up with negligible attrition.

Several limitations warrant mention. First, this was a single-centre cohort of modest size from one outpatient clinic in Romania. Healthcare organization, referral pathways, access to lipid specialists, reimbursement criteria, formulary restrictions, and prescribing patterns for ezetimibe, PCSK9-directed therapies, and fixed-dose combinations vary substantially across European countries. Consequently, the observed treatment-intensity profile and target-attainment rates should not be assumed to represent other national health systems; external validity is strongest for ambulatory settings with similar Central and Eastern European organizational and reimbursement constraints. Multi-centre and multinational validation are needed before broader generalization.

Second, although the full lipid panel was measured at each visit, the pre-specified analytic variable was the guideline-anchored lipid category rather than the continuous concentration. This design preserves clinically decisive treatment thresholds but precludes computation of exact percentage reductions and continuous on-treatment distributions in the primary endpoint. The absolute concentrations, percentage change from baseline, and the ≥50% reduction co-criterion were not formally evaluated. The magnitude of LDL-C lowering should therefore be interpreted at the level of guideline strata, and attainment may be modestly overestimated in patients who met the absolute threshold without also achieving the relative-reduction requirement.

Third, the small number of patients attaining the target within the high- and very-high-risk strata produced data sparsity and quasi-complete separation. Firth penalized-likelihood regression provided more stable estimates than conventional maximum likelihood, but the odds ratios remain imprecise and should be interpreted as indicators of association rather than as exact or causal effect sizes. Larger cohorts are required to estimate stratum-specific effects reliably.

Fourth, medication adherence was not measured objectively using pharmacy-refill data, pill counts, electronic monitoring, or validated adherence instruments. It is, therefore, impossible to quantify how much target non-attainment resulted from physician-level therapeutic inertia, failure to initiate or persist with prescribed therapy, intermittent medication use, or other patient-level behaviour. The low documented prescribing rate of high-intensity and combination regimens supports under-escalation as a plausible contributor, but prescription records do not prove actual exposure, and the relative contributions of prescribing and adherence cannot be disentangled in this dataset.

Fifth, the observational design precludes causal inference, and residual or unmeasured confounding cannot be excluded. Finally, hard clinical endpoints were not captured over this time horizon. The decreases in risk scores represent changes in modelled estimates—partly determined mathematically by improved lipid inputs—and are not direct proof of fewer cardiovascular events. These limitations notwithstanding, the prospective design and concordance of the central target-attainment finding with large European registries support the relevance of the observed implementation gap within comparable clinical settings.

## 5. Conclusions

Over one year of routine-care lipid-lowering therapy, lipid-target attainment improved substantially in this ambulatory cohort, while recalculated cardiovascular risk scores moved in a favourable direction. The score changes reflect updated model inputs and should not be interpreted as direct evidence of reduced cardiovascular events. A pronounced, non-monotonic attainment gap persisted: high- and very-high-risk patients reached their LDL-C targets least often, and higher risk category was associated with non-attainment in a sparse multivariable model whose odds ratios are not precise causal effect estimates. Conservative prescribing intensity was documented, but the absence of objective adherence measurement prevents separation of therapeutic inertia from patient-level medication behaviour. Earlier guideline-concordant escalation, combination lipid-lowering therapy, structured follow-up, and objective adherence assessment may help reduce this implementation gap, while individualized safety monitoring remains appropriate in patients receiving intensive therapy or concomitant antithrombotic treatment.

## Figures and Tables

**Table 1:** Baseline demographic, clinical and biochemical characteristics of the study cohort (*n* = 101).

Characteristic	Value	Unit/*n* (%)
Age, mean ± SD	58.2 ± 7.1	years
Female sex	60	(59.4%)
Urban residence	64	(63.4%)
Diabetes mellitus	39	(38.6%)
Established ASCVD	23	(22.8%)
Arterial hypertension	99	(98.0%)
Family history of premature CVD	44	(43.6%)
Active smoking	37	(36.6%)
Sedentary lifestyle	66	(65.3%)
Elevated lipoprotein(a)	10	(9.9%)
Statin-naïve at baseline	10	(9.9%)
Very-high cardiovascular risk	32	(31.7%)
High cardiovascular risk	32	(31.7%)
Moderate/low cardiovascular risk	37	(36.6%)

ASCVD, atherosclerotic cardiovascular disease; CVD, cardiovascular disease; SD, standard deviation.

**Table 2:** Risk-stratified LDL-C and non-HDL-C target attainment at baseline and 12 months.

Risk Category (n)	LDL-C T0	LDL-C T2	Non-HDL T0	Non-HDL T2
Moderate/low (37)	8%	89%	8%	89%
High (32)	3%	9%	3%	38%
Very high (32)	6%	23%	10%	22%
Whole cohort (101)	6%	43%	7%	52%

Values are the percentage of patients within each stratum meeting the guideline-recommended target. LDL-C targets: <55 mg/dL (very high), <70 mg/dL (high), <116 mg/dL (moderate/low).

**Table 3:** Firth penalized-likelihood logistic regression for LDL-C target attainment at 12 months (T2).

Covariate	Adjusted Association (Firth Model)	*p* Value	Interpretation
Age (per year)	No independent association	>0.05	Not significant
Female sex (vs. male)	No independent association	>0.05	Not significant
Statin–ezetimibe combination (vs. none)	No independent association	>0.05	Not significant
High risk (vs. moderate/low)	OR 0.012 (95% CI 0.002–0.06)	<0.001	Lower odds of attainment
Very high risk (vs. moderate/low)	OR 0.034 (95% CI 0.009–0.12)	<0.001	Lower odds of attainment

CI, confidence interval; OR, odds ratio. The model included age, sex, cardiovascular risk category, and statin–ezetimibe combination therapy. Moderate/low risk served as the reference category. Firth penalized-likelihood regression was used because sparse attainment events in the higher risk strata caused quasi-complete separation in the conventional maximum-likelihood model. The reported odds ratios indicate association and should not be interpreted as precise causal effect sizes; despite penalisation, uncertainty remains substantial because of the limited number of events. Age, sex, and combination therapy were not independently associated with attainment (all *p* > 0.05).

## Data Availability

Deidentified clinical, laboratory, and imaging data supporting the findings of this study are available from the corresponding authors upon reasonable request, subject to institutional data-sharing policies.

## Abbreviations

The following abbreviations are used in this manuscript:

ALT	Alanine Aminotransferase
ASCVD	Atherosclerotic Cardiovascular Disease
AST	Aspartate Aminotransferase
CK	Creatine Kinase
CKD	Chronic Kidney Disease
CKD-EPI	Chronic Kidney Disease Epidemiology Collaboration
CV	Cardiovascular
CVD	Cardiovascular Disease
DIAL	Diabetes Lifetime-Perspective Prediction Model
DM	Diabetes Mellitus
EAS	European Atherosclerosis Society
eGFR	Estimated Glomerular Filtration Rate
ESC	European Society of Cardiology
HDL-C	High-Density Lipoprotein Cholesterol
LDL-C	Low-Density Lipoprotein Cholesterol
LIFE-CVD	Lifetime Cardiovascular Disease Risk Model
LLT	Lipid-Lowering Therapy
non-HDL-C	Non-High-Density Lipoprotein Cholesterol
OR	Odds Ratio
PCSK9	Proprotein Convertase Subtilisin/Kexin Type 9
PCSK9i	Proprotein Convertase Subtilisin/Kexin Type 9 Inhibitor
PREVENT	Predicting Risk of Cardiovascular Disease Events Model
SCORE2	Systematic Coronary Risk Evaluation 2
SMART	Secondary Manifestations of Arterial Disease Risk Score
SMART-REACH	SMART Risk Reduction and Extension of Life Expectancy Model
SD	Standard Deviation
T0	Baseline Assessment
T1	Short-Term Follow-Up (6–12 Weeks)
T2	Long-Term Follow-Up (≥1 Year)
